# Supplementary material for: Thermophilic Exopolysaccharide Films: A Potential Device for Local Antibiotic Delivery
Source: Pharmaceutics. 2023 Feb 7;15(2):557. doi: 10.3390/pharmaceutics15020557 (PMC9960241; doi:10.3390/pharmaceutics15020557)
Supplement: Supplementary file 1 [file pharmaceutics-15-00557-s001.zip › pharmaceutics-2124084-supplementary.pdf]

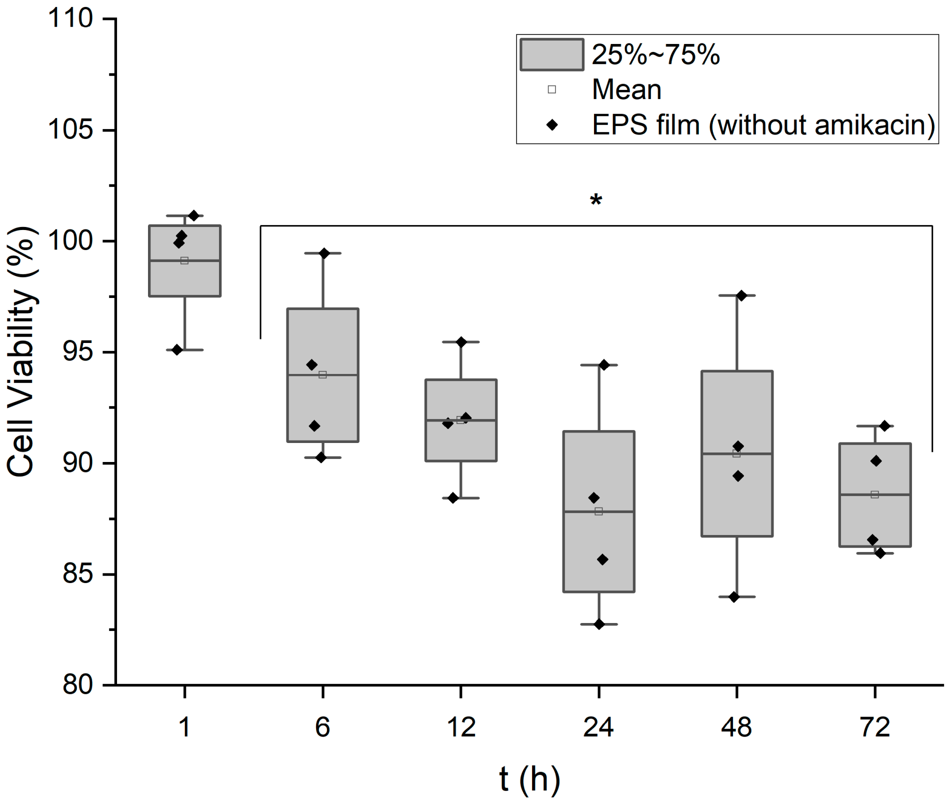

Supplementary Figure S1. Viability results via MTT assay of keratinocytes exposed to an EPS control film (n=4). The control film contained all of the film's reagents minus amikacin. Data was also evaluated for significance using a two-way repeated measures ANOVA and Tukey's test post-hoc. *Versus* untreated cells: * $p < 0.01$.
